# Supplementary material for: Substrate DNA Promoting Binding of Mycobacterium tuberculosis MtrA by Facilitating Dimerization and Interpretation of Affinity by Minor Groove Width
Source: Microorganisms. 2023 Oct 7;11(10):2505. doi: 10.3390/microorganisms11102505 (PMC10609481; doi:10.3390/microorganisms11102505)
Supplement: Supplementary file 1 [file microorganisms-11-02505-s001.zip › microorganisms-2568010-supplementary.pdf]

Table S1. The affinity of MtrA targeted fragments.

Fragment Name	Affinity (KD (M)	Fragment Name	Affinity (KD (M)	Fragment Name	Affinity (KD (M))
Rv2524c	4.62E-08	Rv0469	1.19E-04	Rv0320	1.42E-07
Rv0188	1.74E-03	Rv0073	5.18E-06	Rv2190c	1.34E-06
Rv2412	5.76E-04	Rv0511	2.70E-05	Rv0974c	6.22E-03
Rv3681c	4.41E-05	Rv0937c	1.53E-06	Rv1754c	3.78E-03
Rv0654	5.88E-06	Rv1074c	6.33E-03	Rv0484c	6.19E-05
Rv2162c	7.58E-04	Rv2719c	2.31E-03	Rv2673	1.35E-04
MTB000127	4.60E-04	Rv1683	2.88E-03	Rv3558	2.29E-04
Rv0575c	1.69E-04	Rv1038c	4.31E-06	Rv2434c	8.84E-07
Rv2352c	3.38E-06	Rv1388	1.42E-04	Rv2054	4.11E-03
Rv3857c	1.04E-06	Rv0305c	1.07E-03	Rv1817	1.00E-03
Rv0309	1.78E-04	Rv3209	1.07E-04	Rv3171c	1.20E-05
Rv1425	6.42E-03	Rv0455c	4.46E-03	Rv2351c	1.31E-04
Rv3859c	1.11E-05	Rv2663	7.25E-04	Rv0950c	1.13E-07
Rv3674c	7.95E-06	Rv3330	7.42E-03	Rv0857	1.40E-05
Rv0556	6.91E-04	Rv0172	1.66E-04	Rv1159	8.39E-04
Rv2935	3.09E-04	Rv0508	2.39E-05	Rv3696c	1.26E-03
Rv3246c	9.88E-03	Rv2435c	2.66E-04	Rv1780	1.17E-03
Rv3354	6.11E-04	Rv0676c	2.76E-04	Rv0052	1.22E-05
Rv1896c	4.45E-04	Rv1293	7.72E-05	Rv3644c	6.88E-03
Rv1746	1.88E-03	Rv1315	9.05E-03	Rv3826	4.00E-04
Rv0129c	9.85E-03	Rv2710	8.11E-04	Rv1013	2.24E-07
Rv3809c	9.21E-03	Rv0169	3.42E-04	Rv0402c	8.31E-04
Rv1088c	1.38E-05	Rv1788	8.69E-04	Rv0351	3.88E-06
Rv1396c	2.02E-06	Rv3106	1.12E-04	Rv2252	1.55E-04
Rv3680	1.88E-03	Rv2842c	1.09E-05	Rv3058c	7.29E-04
Rv1481	1.38E-03	Rv1168c	8.11E-04	Rv3248c	7.89E-05
Rv2758c	1.22E-05	Rv0252	1.78E-05	Rv1588c	1.46E-04
Rv0001	1.13E-05	Rv0545c	1.33E-03	Rv2975a	1.18E-05
Rv3416	9.32E-03	Rv1809	5.02E-04	Rv1908c	6.14E-07
Rv2632c	9.13E-03	Rv1511	3.19E-03	Rv1323	1.82E-05
Rv3883c	1.42E-05	Rv3631	4.97E-05	Rv1815	1.24E-07
Rv1458c	1.97E-03	Rv1067c	5.84E-03	Rv3916c	3.73E-03
Rv3197	2.88E-04	Rv1039c	5.36E-03	Rv1791	1.37E-06
Rv1599	6.45E-03	Rv0867c	1.35E-07	Rv1830	3.38E-05
Rv1952	5.29E-03	Rv1915	1.88E-04	Rv2463	4.76E-04
Rv2181	1.12E-05	Rv1796	5.33E-05	Rv2426c	9.59E-04
Rv0507	6.72E-04	Rv2381c	1.48E-04	Rv1635c	4.20E-04
Rv2864c	1.00E-06	Rv1161	8.93E-04	Rv0208c	6.38E-03
Rv2830c	8.53E-03	Rv0916c	9.22E-03	Rv3340	4.41E-04
Rv1523	1.31E-06	Rv2631	6.26E-04	Rv1477	5.77E-06
Rv0947c	7.71E-06	Rv3717	5.51E-05	Rv3159c	7.89E-03

Rv3382c	2.42E-04	Rv1533	6.31E-06	Rv2892c	2.68E-06
Rv0284	4.07E-03	Rv2786c	7.91E-04	Rv2593c	4.02E-05
Rv3910	1.65E-03	Rv2881c	2.66E-04	Rv2941	1.42E-03
Rv3455c	1.17E-03	Rv0822c	1.93E-05	Rv1380	1.00E-03
Rv3924c	8.89E-03	Rv2942	3.55E-04	Rv1272c	8.51E-04
Rv0063	1.76E-03	Rv2492	3.77E-06	Rv2497c	3.20E-07
Rv1047	1.71E-06	Rv3298c	1.65E-06	Rv1036c	1.33E-04
Rv3414	6.68E-04	Rv1772	1.90E-06	Rv0002	8.66E-03
Rv0177	5.84E-04	Rv3488	1.09E-04	Rv0787	1.09E-06
Rv3587c	1.41E-03	Rv2274A	7.22E-05	Rv1759c	5.43E-06
Rv1358	9.28E-06	Rv2522c	6.00E-05	Rv2147c	5.73E-06
Rv3777	2.16E-03	Rv0938	6.71E-05	Rv0930	1.67E-05
Rv0365c	1.02E-05	Rv3500c	1.87E-03	Rv1436	7.78E-05
Rv3762c	8.86E-04	Rv0101	2.22E-04	Rv2794c	2.02E-06
Rv2291	4.75E-06	Rv3037c	5.29E-03	Rv0835	9.68E-05
Rv2145c	6.31E-03	Rv0446c	8.27E-03	Rv2154c	8.29E-03
Rv1497	4.49E-03	Rv1520	5.82E-03	Rv1884c	1.31E-04
Rv0404	3.49E-06	Rv3823c	4.41E-04	Rv3886c	4.88E-04
Rv0244c	1.42E-05	Rv1557	1.38E-05	Rv0015c	1.16E-03
Rv3583c	3.90E-04	Rv2958c	1.01E-04	Rv3004	6.84E-03
Rv3229c	1.46E-04	Rv2485c	7.45E-05	Rv2618	5.49E-05
Rv0790c	5.89E-05	Rv0704	7.31E-04	Rv2988c	2.36E-04
Rv2525c	6.06E-07	Rv1664	7.77E-04	Rv2493	9.36E-04
Rv2590	3.26E-04	Rv0319	4.45E-03	Rv0171	3.83E-04
Rv1404	7.38E-04	Rv0002c	9.22E-03	Rv1116	8.16E-05
Rv3099c	6.30E-04	Rv2123	7.76E-04	Rv3849	9.65E-03
Rv1606	3.59E-03	Rv0533c	1.15E-03	Rv0116c	1.88E-05
Rv1088	5.71E-06	Rv3389c	8.77E-03	Rv1918c	7.90E-08
Rv1006	1.72E-05	Rv2647	1.68E-04	Rv3898c	8.39E-05
Rv3197A	4.78E-05	Rv1373	1.07E-04	Rv1943c	1.13E-05
Rv1801	4.98E-03	Rv1697	7.75E-03	Rv1760	1.04E-03
Rv3682	2.21E-06	Rv0373c	2.05E-03	Rv0207c	1.38E-05
Rv3219	4.77E-03	Rv2742c	3.42E-03	Rv1862	8.54E-05
Rv1688	1.26E-06	Rv3414c	6.08E-04	Rv0666	3.85E-04
Rv0856	3.66E-04	Rv3915	6.44E-03	Rv1750c	1.68E-03
Rv3241c	1.39E-04	Rv2336	1.68E-04	Rv2231c	3.31E-04
Rv0058	7.99E-03	Rv2950c	2.09E-04	Rv3431c	8.17E-05
Rv1791c	1.08E-04	Rv1886c	1.31E-05	Rv1825	4.55E-04
Rv1485	1.58E-03	Rv0451c	1.69E-05	Rv3585	6.49E-04
Rv1700	1.29E-05	Rv3787c	9.20E-04	Rv0986	1.05E-03
Rv3370c	4.82E-03	Rv0179c	1.24E-04	Rv1320c	5.81E-06
Rv1995	1.09E-05	Rv3345c	1.43E-04	Rv1268c	4.18E-06
Rv0983	1.09E-03	Rv0996	2.56E-03	Rv1361c	7.25E-04
Rv1548c	8.38E-05	Rv2450c	1.67E-07	Rv0501	7.19E-04

Rv2065	2.66E-04	Rv1170	2.05E-03	Rv3332	5.52E-04
Rv3779	1.81E-05	Rv1009	1.65E-07	Rv1566c	2.18E-05
Rv1358c	9.18E-04	Rv0348	4.07E-04	Rv0187	1.97E-05
Rv0130	9.52E-03	Rv2342	3.48E-05	Rv1105	3.46E-04
MTB000127c	4.66E-04	Rv0456c	1.37E-04	Rv2847c	3.30E-04
Rv3804c	4.92E-03	Rv2070c	1.09E-04	Rv1011	3.62E-05
Rv3699	1.77E-04	Rv3799c	1.16E-03	Rv2417c	1.21E-03
Rv3059	8.89E-06	Rv2550c	8.08E-05	Rv1433	2.59E-05
Rv2542	1.08E-05	Rv3887c	1.12E-06	Rv2050	2.02E-05
Rv1266c	9.22E-04	Rv2486	5.32E-04	Rv3223c	9.61E-03
Rv0041	5.11E-04	Rv3323c	1.68E-04	Rv1976c	6.12E-05
Rv1360	7.71E-05	Rv0053	1.71E-03	Rv0113	1.52E-04
Rv1221	7.41E-06	Rv1857	9.01E-04	MTB000020	6.77E-05
